# A bioeconomic model for the optimization of local canine rabies control

**DOI:** 10.1371/journal.pntd.0007377

**Published:** 2019-05-22

**Authors:** Aaron Anderson, Johann Kotzé, Stephanie A. Shwiff, Brody Hatch, Chris Slootmaker, Anne Conan, Darryn Knobel, Louis H. Nel

**Affiliations:** 1 USDA National Wildlife Research Center, Fort Collins, CO, United States of America; 2 MSD Animal Health Malelane Research Unit, Malelane, South Africa; 3 Center for Conservation Medicine and Ecosystem Health, Ross University School of Veterinary Medicine, Basseterre, St Kitts and Nevis; 4 Department of Veterinary Tropical Diseases, University of Pretoria, Pretoria, South Africa; 5 Department of Biochemistry, Genetics and Microbiology, University of Pretoria, Pretoria, South Africa; 6 Global Alliance for Rabies Control SA NPC, Pretoria, South Africa; Environment and Sustainability Institute, UNITED KINGDOM

## Abstract

We present a new modeling tool that can be used to maximize the impact of canine rabies management resources that are available at the local level. The model is accessible through a web-based interface that allows for flexibility in the management strategies that can be investigated. Rabies vaccination, sterilization, chemo-contraception, and euthanasia can be specified and limited to specific demographic groups. Additionally, we allowed for considerable complexity in the specification of management costs. In many areas, the costs of contacting additional dogs increases as management effort increases, and this can have important strategic implications. We illustrated the application of the model by examining several alternative management strategies in an area of Mpumalanga Province, South Africa. Our results based on this dog population suggested that puppies should be vaccinated and sterilization would not be optimal if the spatial extent of management is not large (and perhaps not even then). Furthermore, given a sufficient budget, it was evident that vaccination campaigns should be repeated annually.

## Author summary

Rabies transmitted by dogs remains a threat in much of the developing world. Although infection in humans is preventable with pre-exposure or post-exposure prophylaxis, management and elimination of the disease in dogs is the only definitive way to eliminate human risk and avoid the high costs of human treatment. In this article, we present and apply a model of canine rabies management that can be used to maximize the benefits of management resources. We also developed a web application front end for our model so that managers can access it from any computer with an internet connection. Critically, our model can help answer complex strategic questions surrounding vaccine application, population control, campaign timing, and budget allocation. We demonstrated application of the model in a region of Mpumalanga Province, South Africa, and our findings suggested that puppies should be vaccinated, adding female sterilization to vaccination campaigns is not optimal, and campaigns should be repeated annually.

## Introduction

The World Health Organization estimates that about 59,000 people die from rabies each year [1]. Although the threat of infection is relatively low compared to some other diseases, rabies deserves attention because infection in humans is easily prevented with pre-exposure and post-exposure prophylaxis (PEP). Additionally, low-cost and effective vaccines are available for managing and eliminating the disease in domestic dogs, the primary source of human exposure in much of the developing world. Successful management of the disease has been demonstrated in many developed countries where PEP is readily available and vaccination of dogs is common practice. For example, the United States was declared canine rabies free in 2007, after extensive public education and mass vaccination of dogs [2]. Much of Western and Central Europe is also free from the disease, and many countries in Latin America have made substantial progress in recent years [3–5]. In addition to these successes, a collaborative global strategic plan has been developed by the World Health Organization (WHO), the Food and Agriculture Organization of the United Nations (FAO), the World Organisation for Animal Health (OIE), and the Global Alliance for Rabies Control (GARC). The goal of this plan (“Zero by 30”) is to eliminate all human deaths from dog transmitted rabies by 2030 [6, 7].

Nearly all human deaths from canine rabies occur in Africa and parts of Asia, where several obstacles to successful management persist. These obstacles include the number of inaccessible dogs, the inability or unwillingness of owners to bring dogs for vaccination, lack of information about rabies, lack of surveillance and diagnostics, and insufficient resources for veterinary services [8]. In many regions, a lack of education about the need for PEP and an inability to access PEP are also sources of human mortality. Regardless of the particular impediments to successful management, some fundamental constraints can be found in the low priority given to rabies and the insufficiency of resources allocated for its control and elimination. These issues go hand in hand with the fact that public health in general is in a poor state and under significant pressure in much of the developing world. In the case of rabies, many regions lack the basic infrastructure and institutions necessary for effective management. Successful dog vaccination campaigns require functioning transportation and communications infrastructure, and widespread PEP availability requires the existence of clinics or hospitals that are accessible to both the urban and rural populations of countries.

Our focus in this paper is based on two observations. First, the elimination of human exposure in the developing world results from the elimination of the disease in dogs. Eliminating the disease in dogs provides ongoing benefits by avoiding the relatively high and unending costs of human treatment. Furthermore, in any population, there will always be people who are unwilling or unable to obtain PEP in response to a potential exposure. Thus, elimination of the disease in dogs will reduce human mortality even if access to PEP is widespread. Our second observation is that planning and funding for canine rabies management are often haphazard. Coordinated international efforts are rare, and even efforts within a single country may not be well-coordinated. With these observations in mind, and given the near universal lack of sufficient management resources, our goal was to develop a tool that can be used to maximize the impact of whatever canine rabies management resources are available at the local level.

The tool we have developed is a bioeconomic model that can be accessed through a web-based graphical user interface (www.bioeconmodel.com) as well as complementary script-based model and Jupyter Notebook implementations. The model is an individual-based, stochastic simulation model that explicitly accounts for the links between management effort, management cost, and biological outcomes. Additionally, our objective was to construct a model that (1) accounts for population and disease dynamics, (2) allows vaccination, permanent sterilization, temporary contraception, and removal, (3) allows strategies to vary temporally and demographically, (4) allows combination strategies, and (5) is flexible enough to allow parameterization for many different canine rabies management scenarios. Although all our code is freely available (https://github.com/anderaa/bioecon) and can be modified by a user if desired, our model can be used in applied settings by users without computer programming experience. The web-based framework was chosen over an installable desktop application because it only requires an internet connection and a web browser. The performance of the model is not affected by the speed of the internet connection or the hardware of the user’s computer because all computations are performed on a remote server. Additionally, the model will run on any operating system, even those that are substantially outdated.

To illustrate application of the model, we investigated several aspects of rabies management in free-ranging dog populations in South Africa. Specifically, we investigated the optimality of puppy vaccination and of combining sterilization and vaccination to minimize the impacts of the disease. The manuscript proceeds with a description of the model and the details of its various mechanisms. This is followed by the presentation of the case study. We provide details of the process of parameterizing the model for a region in South Africa and examining alternative management strategies within this region. After presenting and analyzing the results of our application and performing a sensitivity analysis, we close with a discussion of the various ways the model can be used and the shortcomings that users should be aware of.

## Methods

### Model overview

There are several key characteristics of the model. First, the model tracks individual dogs and their traits through time. This is performed via a matrix that contains a row for each individual and a column for each trait associated with individuals (Table 1). Second, the model operates on a daily time step. This minimizes bias that results from discrete time steps, and allows the model to more precisely consider management efforts that vary temporally. Third, many of the processes that occur in the model are stochastic. A stochastic model provides important benefits because it allows a user to examine the tradeoffs between management costs and the certainty with which a management goal is successfully achieved. Fourth, the model allows nearly any combination of vaccination, fertility control, and removal, and these treatments can be demographic-specific. Finally, the model separates the cost of capturing or contacting dogs and the cost of applying the treatment. Notably, the user can easily specify a non-linear relationship between the cost of capturing dogs and the number of dogs captured. The separation of costs and ability to specify non-linear capture costs are critical to a proper understanding of the economics of management, and we are unaware of the existence of any other model of rabies management that specifically addresses this factor.

**Table 1:** Columns of the population matrix.

Trait	Notes
age	integer–days
puppy	boolean–yes/no
adult	boolean–yes/no
female	boolean–yes/no
sterilized	boolean–yes/no
contracepted	boolean–yes/no
duration of contraception	integer–days
booster vaccine received	boolean–yes/no
exposed	boolean–yes/no
infective	boolean–yes/no
timeLimitExposed	float—days
timeLimitExposed	float—days
time infective	integer–days
immune	boolean–yes/no
month	integer–month number

**Fig 1:**
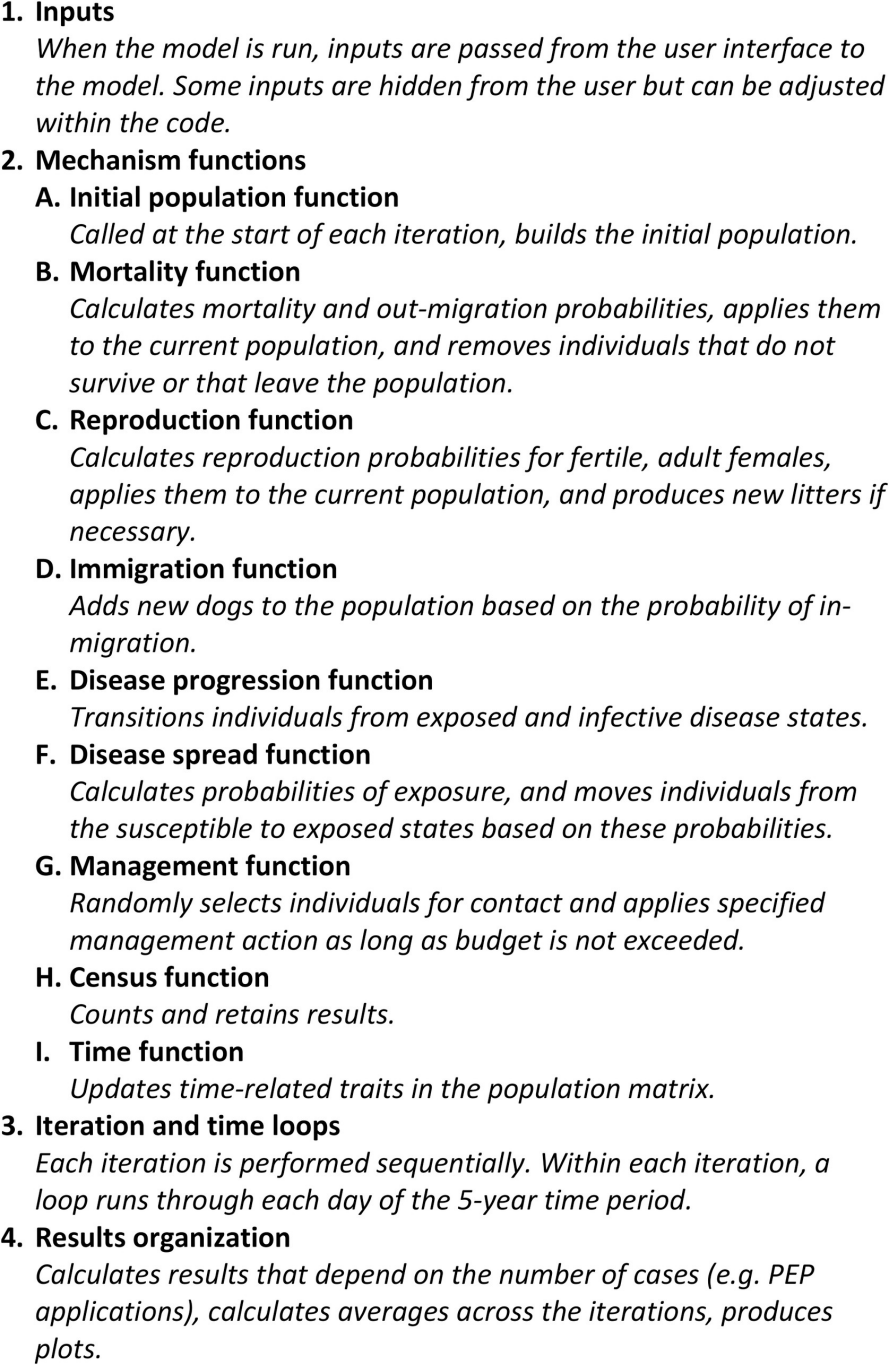
Outline of code in the model script.

We are acutely aware of the perception that many individual-based models are not amenable to fast and thorough investigation and thus considered black boxes. We have taken a number of steps to mitigate this concern here. Our model is written in the R language [9] using the Shiny framework for the web application. R was chosen over other languages because its use by researchers is common and growing, it is free and open-access, and the code is relatively easy to read. Additionally, we have also structured the code in a way that facilitates easy understanding. The code for the web app model consists of two scripts, one that creates the user interface and one that defines the model. The script that defines the model consists of four main sections (Fig 1). All code has been carefully annotated to ease understanding. Additionally, major mechanisms within the model are contained in their own functions. While this structure eases understanding, it also makes the code modular and easy to modify. If a user wants to change a mechanism, a single function can be changed without the risk of interfering with other mechanisms. Finally, there were a number of situations where we faced a choice of employing code that was faster or employing code that was easier to understand. In most cases, we chose the latter. In the following sections, we provide brief descriptions of the major mechanisms of the model.

### Mortality and reproduction processes

Non-rabies mortality is caused by two mechanisms. First, a user-specified, annual mortality rate is converted to a daily mortality probability for puppies, juveniles, and adults. Individuals in the population face this probability on each day, and random draws determine their fates. Additionally, if the abundance exceeds the user-specified carrying capacity after probabilistic mortality has occurred, individuals are removed from the population (with probabilities proportional to their daily mortality probabilities) until carrying capacity is reached.

Reproduction is governed by a user-specified probability that a fertile adult female has one litter in a year, as well as average litter size. Additionally, the user can specify certain months in which litters are more likely. This is performed with month check boxes and a parameter that specifies the fraction of all litters during a year that occur in the selected months. The model takes the selected months and fraction of litters born during those months and calculates, for each fertile adult female, the probability of producing a litter during each month of the year. Finally, random draws determine the number of litters produced each month, and new puppies are added to the population.

### Disease introduction

In some cases, canine rabies management may only occur in response to a known outbreak. In other cases, management may be an ongoing attempt to minimize the threat posed by a potential introduction. Our model can be used to investigate either type of management. Rabies can be introduced at any time during the five-year simulation period. Additionally, the user can specify the number of dogs that are exposed during the introduction event, as well as the number of sequential months that introductions occur. This arrangement allows the user to investigate management that is implemented before or after an introduction occurs. It also allows the user to effectively provide a period of time before disease introduction that allows demographics to equilibrate.

### Disease transmission

We assume that the number of bites per rabid dog per day follows a negative binomial distribution. Thus, on each day that at least one rabid dog exists, random draws determine the total number of bites that rabid dogs inflict on other dogs. These bites are allocated across the population randomly. Susceptible dogs that receive a bite face a user-specified probability of infection. If a random draw implies infection, the dog is moved from the susceptible state to the exposed state. Dogs remain in the exposed and infective states for periods of time determined by random draws from gamma distributions. Although these values are not adjustable in the user interface, they are clearly marked and easily adjusted within the code. There are also several additional transmission-related inputs that are adjustable within the code. By default, the probability of survival is zero, but this can be adjusted so that some small percentage of dogs recovers with immunity. Additionally, the number of dogs immune (either from recovery or vaccination) in the initial population can be specified.

We acknowledge that heterogeneity in the number of bites per rabid dog could be related to other characteristics of the dog (e.g. age, sex, contact cost). We chose not to account for these types of relationships because we lack that necessary data in our application and we assume that most users would also lack this type of data. Furthermore, it would be difficult to cleanly incorporate specification of these relationships in the user interface. For applications that have sufficient data, these relationships could be accounted for by adjusting the model code.

### Disease impacts

The user can specify two impacts on human health: PEP applications and mortality. To enable estimation of these impacts, the user first specifies the number of bites per day by rabid and non-rabid dogs. Then the probability of PEP applications for each bite type, as well as the cost of each PEP application, is specified. Finally, the probability of human death, given a bite from a rabid dog, is specified. Given these inputs and the number of rabid and non-rabid dogs on each day of the simulation period, the model calculates the number of PEP applications and human deaths on each day.

### Management costs

**Fig 2:**
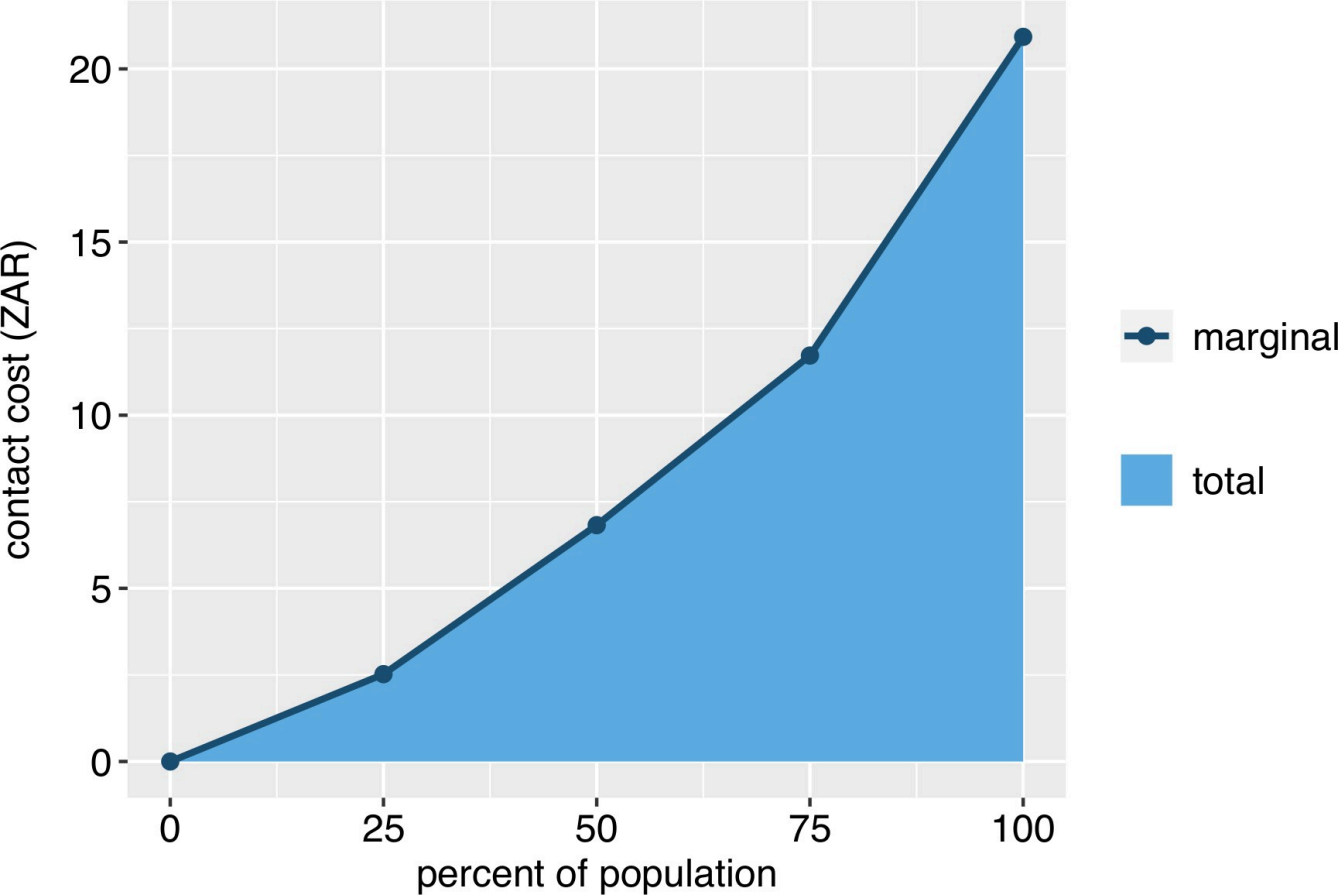
The stepwise-linear function for capture or contact costs used in our application.

We define marginal strategy cost as the cost of applying a chosen strategy to an additional dog. Furthermore, the marginal strategy cost is the sum of the marginal treatment cost and the marginal contact (i.e. capture) cost. The separation of treatment costs and contact costs and the ease with which a user can specify a non-linear marginal contact cost function are important characteristics of the model. The model assumes that marginal treatment (i.e. vaccination, sterilization, contraception, removal) costs are constant as the number of dogs treated varies. These are specified on a per-dog basis and sterilization and contraception costs are sex-specific. Marginal contact costs are derived by estimating the cost of contacting or capturing 25%, 50%, 75%, and 100% of the population (Fig 2). Because the resulting function may be non-linear (technically piecewise-linear), the marginal cost of the chosen strategy may be both an increasing and non-linear function of the number of dogs treated. This contrasts with most existing models of rabies control, which only incorporate constant costs of treatment.

It is intuitive that marginal strategy costs are typically not constant. Some dogs are quite easy to capture or contact, while other dogs are very difficult and therefore consume more resources to be captured. This can have important implications for optimizing management strategies. For example, a manager choosing a mix of vaccination and fertility control may choose to devote all resources to vaccination if contact costs are ignored. Alternatively, if contact costs are considered, and if those costs sharply increase beyond a certain point, it may be more beneficial to capture fewer dogs and instead apply vaccination *and* fertility control to the dogs that do get captured. As an example, suppose that contact and treatment costs are such that a manager can a) contact and vaccinate 80% of the population or b) contact, sterilize, and vaccinate 40% of the population. If marginal contact costs instead increased more sharply, the percentages of the population contacted in the two options would be more equal given the same budget. A variety of similar tradeoffs exist. As a result, when there is a choice between concentrating resources on a specific treatment, demographic group, or time period or, instead, spreading resources more broadly, it is imperative that the increasing, non-linear nature of contact and strategy costs is accounted for.

Although we believe that the ability to account for non-linear strategy costs is an important part of the model, we also recognize that sufficient data must be available to properly estimate such a relationship. As a result, the model can also accommodate constant capture and strategy costs. This can be done by setting all points that produce Fig 2 to the average cost of contacting a dog, or by setting contact costs to zero and adding the average cost of contacting a dog to the treatment costs. Furthermore, we recognize that management often involves a mix of different types of vaccination campaigns (e.g. door-to-door, central point). Although we do not allow the user to explicitly specify this, it is indirectly implied by the way contact costs are specified. Suppose, for example, that approximately 50% of dogs can be contacted via central point campaigns, 25% via a door-to-door campaign, and 25% are virtually unreachable. The user could account for this by specifying the appropriate contact cost structure. In the case of unreachable dogs, the user would simply input some arbitrarily high cost of contacting the final 25% of dogs to ensure they are never contacted.

### Management budget

The user specifies a management budget for each year of the 5-year simulation period to be allocated to the chosen strategy. Note that the management budget does not include spending on PEP because we assume that the funding mechanisms are typically different. The annual budget is then spread over the days of the months that management will occur in. Each day, the management function checks for a non-zero budget and sequentially captures dogs and carries out the specified treatment(s). To ensure that the contact costs correspond to Fig 2, all dogs entering the population are assigned one of the four marginal contact costs with equal probability. On a given day, the management function checks for any dogs in the lowest marginal cost category that have not been captured during the current year. If there are dogs that meet these criteria, one dog from this group is randomly selected and treated and costs are recorded. If there were no uncontacted dogs in the lowest marginal cost category, the function repeats the process for each sequentially higher marginal cost category until an uncontacted dog is found or until the highest category is checked. This entire process continues as long as the available budget has not been exceeded or until all dogs have been contacted.

### Application

Canine rabies remains endemic in South Africa, and is relatively common in the KwaZulu-Natal, Eastern Cape, Mpumalanga, Free State and Limpopo Provinces [10]. From 2012 to 2015, 30 human cases were reported, although this likely represents an underestimate of the true number [10, 11]. Historically, dogs have been responsible for the vast majority of human cases in South Africa, and most victims have been children under 10 years of age [12]. Although the number of reported human cases each year is relatively small, many people receive PEP. The economic burden of PEP is substantial, amounting to R70 million per year, much of which falls on an already-stressed public healthcare system [11].

Managers tasked with minimizing canine rabies in South Africa (and elsewhere) face a variety of strategic choices that include the type and timing of vaccination campaigns. Central-point vaccination campaigns can be advantageous because they rely on owners to bring dogs for vaccination. As a result, contact costs are relatively low. In some areas, dogs will often be brought by children, so operating these campaigns when school is not in session will further increase coverage. However, in areas with high abundance of free-ranging or semi-owned dogs, this type of campaign may be less useful and more active contact and capture efforts may be required.

In addition to the type and timing of campaigns, managers must also decide the amount of resources to allocate to a specific area and how often to repeat campaigns. It is typically recommended that managers attempt to achieve 70% vaccination coverage, but it is often unclear what sort of funding will be required to reach this objective. Furthermore, there is high turnover in most free-ranging dog populations, and vaccination coverage declines rapidly. Quantifying this rate of decline and understanding how often campaigns must be repeated to maintain coverage would assist managers in planning future vaccination.

Besides these broader questions related to resource requirements and vaccination campaigns, there are a variety of more specific choices related to what to do with dogs that are captured or contacted. These questions include which demographic groups to vaccinate, whether to give booster vaccinations to previously vaccinated dogs, and whether there is a role for population management and fertility control. Although our objective was to design a tool that would help managers answer any of these questions, we limited the application that we present here to two important strategic questions.

The first question that we addressed is whether it is beneficial to vaccinate puppies (<90 days old). Historically, puppies have sometimes been excluded from mass vaccination campaigns on the grounds that their immature immune systems may not reliably respond to the vaccine. However, recent evidence (e.g. [13]) suggests that puppies do reliably respond to vaccination, and puppy vaccination is common practice [14].

Barring differences in vaccine response, given a choice between vaccinating a puppy or an adult, the adult would be preferred due to the lower mortality rate of adult dogs. A puppy is more likely to exit the population during a given time period, and any vaccination resources devoted to that dog would be wasted if the dog died or otherwise left the population. However, there are certain conditions under which vaccinating puppies would unquestionably be desirable. If excess vaccination resources are available after all juvenile and adult dogs have been vaccinated, and the goal is to minimize disease (number of dog cases or human risk), then puppy vaccination is clearly desirable. Even if insufficient vaccination resources are available for juvenile and adult dogs, the nature of contact costs may make puppy vaccination desirable. This would occur if the marginal cost of contacting additional dogs increased sharply enough. In this situation, a manager would face a choice between vaccinating many puppies or only a few additional non-puppies. Thus, even if vaccinating a single puppy does not provide the same benefits as vaccinating a single adult, the fact that many more puppies can be vaccinated may lead to an optimal strategy that includes puppy vaccination. A final consideration would be how the population responds to a rabies outbreak. Adult vaccination may lead to less adult mortality and a higher reproduction rate, which could lead to longer-lasting outbreaks or a population that recovers faster and is therefore more susceptible to subsequent rabies introductions.

The second question we sought to answer is whether female dogs that are contacted during vaccination efforts should be sterilized at the same time. There is substantial debate about the role of sterilization and other population management strategies within dog vaccination programs [15]. The OIE recommends dog population control as an integral part of vaccination programs [16], but [17] offer an opposing view based on the lack of evidence that rabies transmission depends on dog density. Our interest in examining female sterilization is based on evidence that suggests female sterilization is much more effective than male sterilization at reducing abundance [18, 19].

The answer to the question of whether and to what extent female sterilization should be integrated into vaccination campaigns involves tradeoffs similar to those of the puppy vaccination question. If sufficient resources are available to vaccinate all dogs in the population, then any additional resources devoted to sterilization will reduce the need to vaccinate in the future because population growth will slow. In the absence of sufficient resources to vaccinate all dogs, a manager will face a choice between vaccinating relatively more dogs or vaccinating fewer dogs but also sterilizing the females that are contacted. Although vaccination will immediately reduce rabies cases and sterilization will not, sterilization might still be preferred if the effect on population growth makes the population substantially less susceptible to disease, makes high levels of vaccination coverage much less costly in the future, or increases the proportion of vaccinated dogs by slowing population turnover. Furthermore, if marginal contact costs increase as more dogs are captured, it may have been possible for the manager to sterilize many dogs rather than vaccinate only a few additional dogs.

### Study area

**Fig 3:**
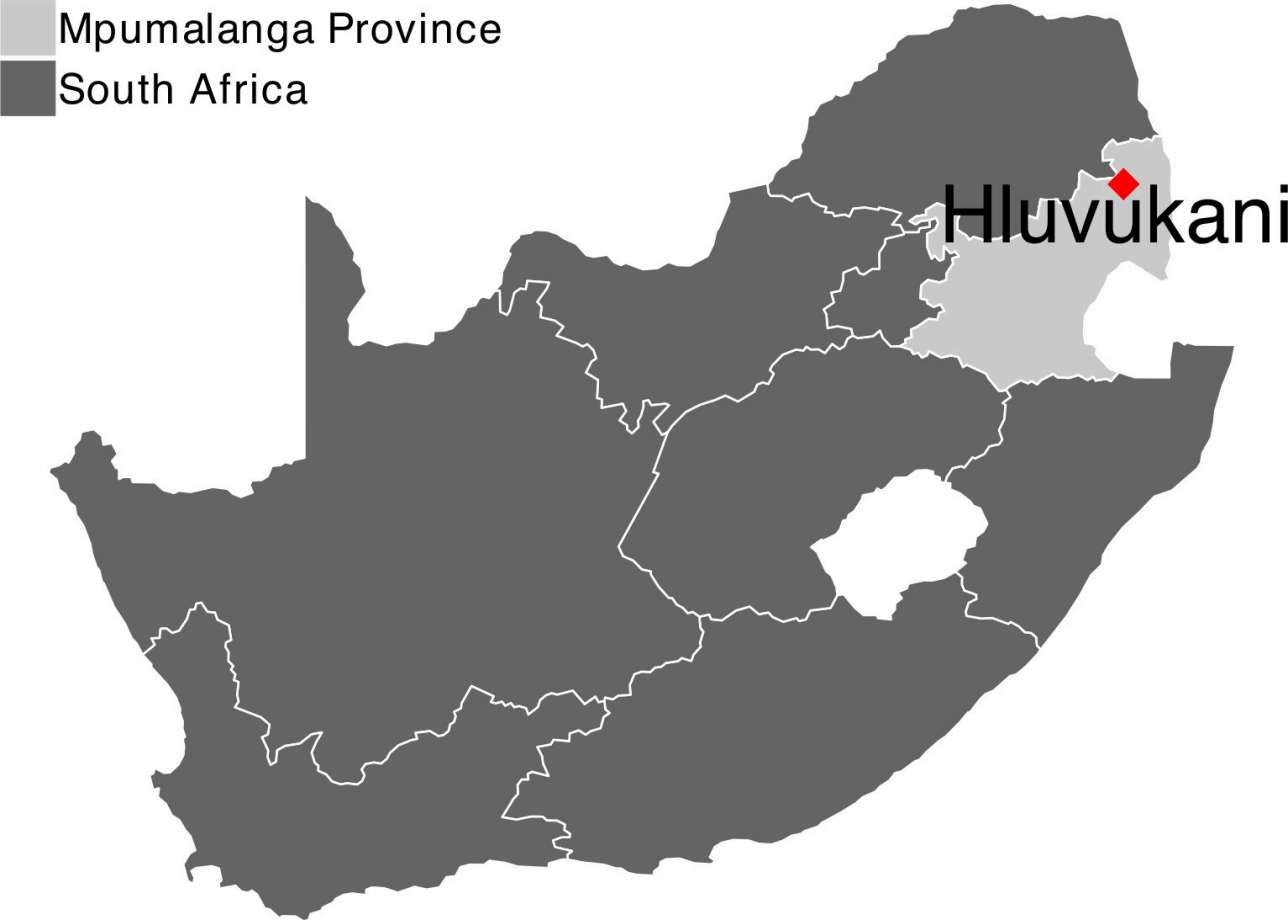
Location of our study area for the health and demographic surveillance system. Map produced using R gglpot and data from https://gadm.org/download_country_v3.html.

In 2011, we established a health and demographic surveillance system (HDSS-Dogs) in a population of owned, largely free-roaming dogs in a low-income community in the village of Hluvukani, Mpumalanga Province, South Africa (Fig 3). We defined a demographic surveillance area (DSA) using natural and artificial boundaries, and monitored all of the approximately 2,500 households in the DSA through regular visits, every five to six months. In each household, we collected data on entry and exit events of owned dogs (birth, death, in- and out-migration). Dogs that entered this population were uniquely and permanently identified by subcutaneous implantation of a radio frequency identification microchip, or through photo identification if they could not be handled. Dates of events were estimated by owners, with uncertainty reflected by a lower and upper estimate of the time since the event. We considered the midpoint between the estimates to be the estimated event date. At each visit, we recorded the rabies vaccination status of new dogs, and updated the vaccination history of dogs in the household since the previous visit. To date, the HDSS-Dogs has provided data on the lives of over 3,000 dogs in the DSA. Further details of the study area and dog population are provided in [20].

### Parameter estimation

The data collected from the study area provided the basis for many of the parameters used in the model (Table 2). Although data collection began in 2011, reproduction and mortality parameter estimations were based on data collected from January 2012 to January 2017 to minimize any bias introduced by irregular reporting early in the study period. Data collection was less consistent in November and December of 2016, and it is possible that some events were missed during these months. However, this was not apparent in an examination of the summary statistics, and we preferred to include the additional data so that our parameters could be based on a longer time series. Although a full accounting of the population was not complete by January 2012, it was not needed for these parameters. However, a more complete accounting of the population was needed to estimate abundance and immigration parameters. Thus, we based these parameters on data collected from January 2013 to January 2017. Daily mortality probabilities were estimated using binary-outcome probit models estimated via maximum likelihood. The daily predicted probabilities for each age class were then annualized for use in the model. There were many cases when a dog exited the population for unknown reasons. As result, we chose to include all exit events in the mortality analysis. Thus, we set out-migration to zero in our application of the model, and our mortality parameters reflect mortality as well as all other types of exit events.

**Table 2:** Model parameters.

variable name	description	location	default	source	notes
***simulation inputs***					
simulationYears	Number of years in the simulation	code	5	-	-
iterations	Number of iterations	ui	5	-	-
***initial population inputs***					
initialPopSize	Initial abundance	ui	463	HDSS data	mean abundance over observation period
initialFracAdult	Fraction of initial population that are adult	ui	0.61	HDSS data	mean over observation period
initialFracPup	Fraction of initial pop. of non-adults that are puppies	ui	0.33	HDSS data	mean over observation period
initialFracFemale	Fraction of initial population that are female	code	0.38	HDSS data	mean over observation period
initialFracImmune	Fraction of initial population that are immune	code	0	-	-
initialFracContra	Fraction of initial pop. that have been contracepted	code	0	-	-
initialFracVacc	Fraction of initial pop. that have been vaccinated	code	0	-	-
initialFracSter	Fraction of initial population that have been sterilized	code	0	-	-
***population model inputs***					
maxJuvAge	Day age at which juveniles transition to adult	code	299	expert opinion	approximate age of sexual maturity
maxPuppyAge	Day age at which puppies transition to juveniles	code	89	expert opinion	approximate age of dispersal from litter
maxAge	Maximum possible age of a dog in days	code	4000	expert opinion	-
carryingCap	Carrying capacity	ui	577	HDSS data	maximum over observation period
pupAnnMortProb	Annual mortality probability of a puppy	ui	0.9	HDSS data	estimated from data
juvAnnMortProb	Annual mortality probability of a juvenile	ui	0.63	HDSS data	estimated from data
adultAnnMortProb	Annual mortality probability of an adult	ui	0.32	HDSS data	estimated from data
emigrationProb	Annual prob. of non-mortality exit from the pop.	ui	0	-	mortality probability incorporates non-mortality exit
immigrantDogs	Number of dogs moving into the population annually	ui	131	HDSS data	annual average over observation period
expectedLittersPFY	Expected litters per fertile female per year	ui	0.31	HDSS data	mean over observation period
meanLitterSize	Mean litter size	code	4.4	HDSS data	mean over observation period
femalePupProb	Fraction of puppies that are female	code	0.38	HDSS data	calculated from data
fractionBirthPulse	Fraction of litters born during the birth pulse	ui	0	HDSS data	none observed in data
birthPulseVector	Months that define the birth pulse	ui	[False, …, False]	HDSS data	none observed in data
***disease model inputs***					
monthsOfPressure	Number of sequential months of introduction	ui	0	-	-
dogsPerMonthExposed	Dogs per month exposed during introduction	ui	0	-	-
monthInitIntroduction	Month of initial introduction	ui	0	-	-
exposedTimeShape	Days in exposed state shape	code	1.08549	Hampson et al. 2009[21]	-
exposedTimeRate	Days in exposed state rate	code	0.04920		
infectiveTimeShape	Days in infective state shape	code	2.83179	Hampson et al. 2009[21]	-
infectiveTimeRate	Days in infective state rate	code	0.91936		
survivalProb	Survival probability	code	0	assumed	-
bitesPerRabidMean	Bites per rabid mean	ui	2.15	Hampson et al. 2009[21]	-
bitesPerRabidShape	Bites per rabid shape	code	1.33	Hampson et al. 2009[21]	-
probInfectionFromBite	Probability of infection from bite	code	0.49	Hampson et al. 2009[21]	-
***disease impact inputs***					
bitesPerNonRabid	Mean daily bites from a non-rabid dog	ui	0.00006	Hampson et al. 2015[22], SACAC 2011[23]	Calculated from Hampson et al. 2015 and est. dog pop.
bitesPerRabid	Mean daily bites from a rabid dog	ui	0.02252	Hampson et al. 2015[22], SACAC 2011[23]	Calculated from Hampson et al. 2015 and est. dog pop.
PEPperNonRabidBite	PEP applications per bite from non-rabid dog	ui	0.991	Hampson et al. 2015[22], SACAC 2011[23]	Calculated from Hampson et al. 2015 and est. dog pop.
PEPperRabidBite	Number of PEP applications per bite from rabid dog	ui	0.991	Hampson et al. 2015[22], SACAC 2011[23]	Calculated from Hampson et al. 2015 and est. dog pop.
costPerPEP	Cost per person treated with PEP	ui	R754.92^1^	expert opinion	-
lifeLossPerRabidBite	Mean human deaths from a rabid dog bite	ui	0.19	Hampson et al. 2015[22]	-
***management inputs***					
vaccineCost	Cost to vaccinate one dog, excluding contact cost	ui	R2.426	expert opinion	-
contraceptionCostFemale	Cost to contracept one female, excl. contact cost	ui	R150	assumed	unused in current application
contraceptionCostMale	Cost to contracept one male, excluding contact cost	ui	R150	assumed	unused in current application
sterilizationCostFemale	Cost to sterilize one female, excluding contact cost	ui	R300	expert opinion	-
sterilizationCostMale	Cost to sterilize one male, excluding contact cost	ui	R200	expert opinion	-
euthanasiaCost	Cost to euthanize one dog, excluding contact cost	ui	R30	assumed	- unused in current application
timeVaccineEffective	Years that the vaccine remains effective	ui	2	Hampson et al. 2007[24]	-
timeBoosterEffective	Years that vaccine remains effective after booster	ui	3	expert opinion	-
timeContraEffectiveMales	Years that male contraceptive remains effective	ui	2	assumed	unused in current application
timeContraEffectiveFemales	Years that female contraceptive remains effective	ui	2	assumed	unused in current application
contactCost25	Cost of contacting 25% of the dogs in the population	ui	1019.09	MVC data	-
contactCost50	Cost of contacting 50% of the dogs in the population	ui	2757.3	MVC data	-
contactCost75	Cost of contacting 75% of the dogs in the population	ui	4735.89	MVC data	-
contactCost100	Cost of contacting all of the dogs in the population	ui	8453.7	MVC data	-
mgtMonthVector	Vector of months that management will occur	ui	[0,.., 0]	-	-
annualBudget	Vector with elements for each of 5 years	ui	[0,.., 0]	-	-
boosterGiven	Booster given to already vaccinated dogs	ui	True	-	-
vacc<demographic><sex>	Dogs in this group vaccinated if contacted	ui	False	-	-
ster<demographic><sex>	Dogs in this group sterilized if contacted	ui	False	-	-
contra<demographic><sex>	Dogs in this group contracepted if contacted	ui	False	-	-
euth<demographic><sex>	Dogs in this group removed from pop. if contacted	ui	False	-	-

^1^ 1 ZAR = 0.073 USD as of January 2019

Three reproduction parameters were estimated from the HDSS data: expected litters per female per year, mean litter size, and the fraction of puppies that are female. These were simple calculations from the data collected from January 2012 to January 2017. We also investigated the data for evidence of a seasonal variation in reproduction, but no strong evidence was found. As a result, we chose not to include any birth pulse in our application of the model. In addition to these reproduction parameters, we also calculated the average number of dogs moving into the population over a four-year observation period. We then annualized this result for use in the model.

The parameters that govern disease transmission and progression were based on published estimates (Table 2). However, parameters and settings that define disease introductions were based on specific assumptions that we have made for our application. Because we wanted to investigate both pre-emptive and reactive management strategies, we assumed that rabies is introduced by a single rabid dog (perhaps from outside the area occupied by the modeled population) at the beginning of the third year.

### Cost estimates

We set the cost of dog treatment (vaccination, sterilization, contraception, and euthanasia) and the cost of human PEP based on recommendations of experts in South Africa (personal communication, Dr. Johann Kotzé). The cost of contacting dogs was estimated based on data we collected during mass vaccination campaigns in 37 different villages in the Bushbuckridge and Mbombela municipalities during 2015. Specifically, we recorded labor hours, wages, and kilometers driven from efforts that included both central-point and door-to-door campaigns. Typical of vaccination efforts in the region, central-point campaigns were used to contact approximately the first 25% of dogs, with door-to-door campaigns accounting for the remainder. Vaccination campaigns in this area were concentrated in the months of April, June, and September. In our application, we assumed all campaigns occur in April.

### Human impacts

The number of human bites was based on an estimate of approximately 423 bites per 100k human population [22]. From this we calculated 213,583 total bites based on a human population of just over 50 million. [22] also provided an estimate of the probability that a bite is from a rabid dog of 0.111. We leveraged this information to split total bites into rabid and non-rabid. Following [22], we then calculated the incidence of rabies in the dog population as

$$Incidence=0.00215{\left(1-0.63\right)}^{1.912}=0.0003$$ (1)

where 0.63 was the average vaccination coverage in South Africa. Finally, given a total dog population of 8,897,064 [23] and our total number of bites, we estimated the number of human bites per rabid dog per day to be 0.02252 and the number of human non-rabid bites per dog per day to be 0.00006. Unfortunately, we lacked data to relate the probability of receiving PEP to the rabies status of the dog. As a result, we assumed a probability of 0.991 of receiving PEP as a result of a dog bite [22]. To the extent that this is inaccurate, the model would underestimate the number of PEP applications given non-zero disease prevalence. However, all other results of the modeling exercises were unaffected. Finally, based on communication with the National Institute for Communicable Disease, we assumed per-person PEP costs to be R754.92 (1 ZAR = 0.073 USD as of January 2019) based on the prevailing retail price of the vaccine in South Africa in 2017 (4 x R188.73) Due to the scarcity of rabies immunoglobulin (RIG) in most countries of the developing world, the cost of RIG was not included, even though the administration of RIG is recommended for category 3 exposures [25].

## Results

### Baseline

**Fig 4:**
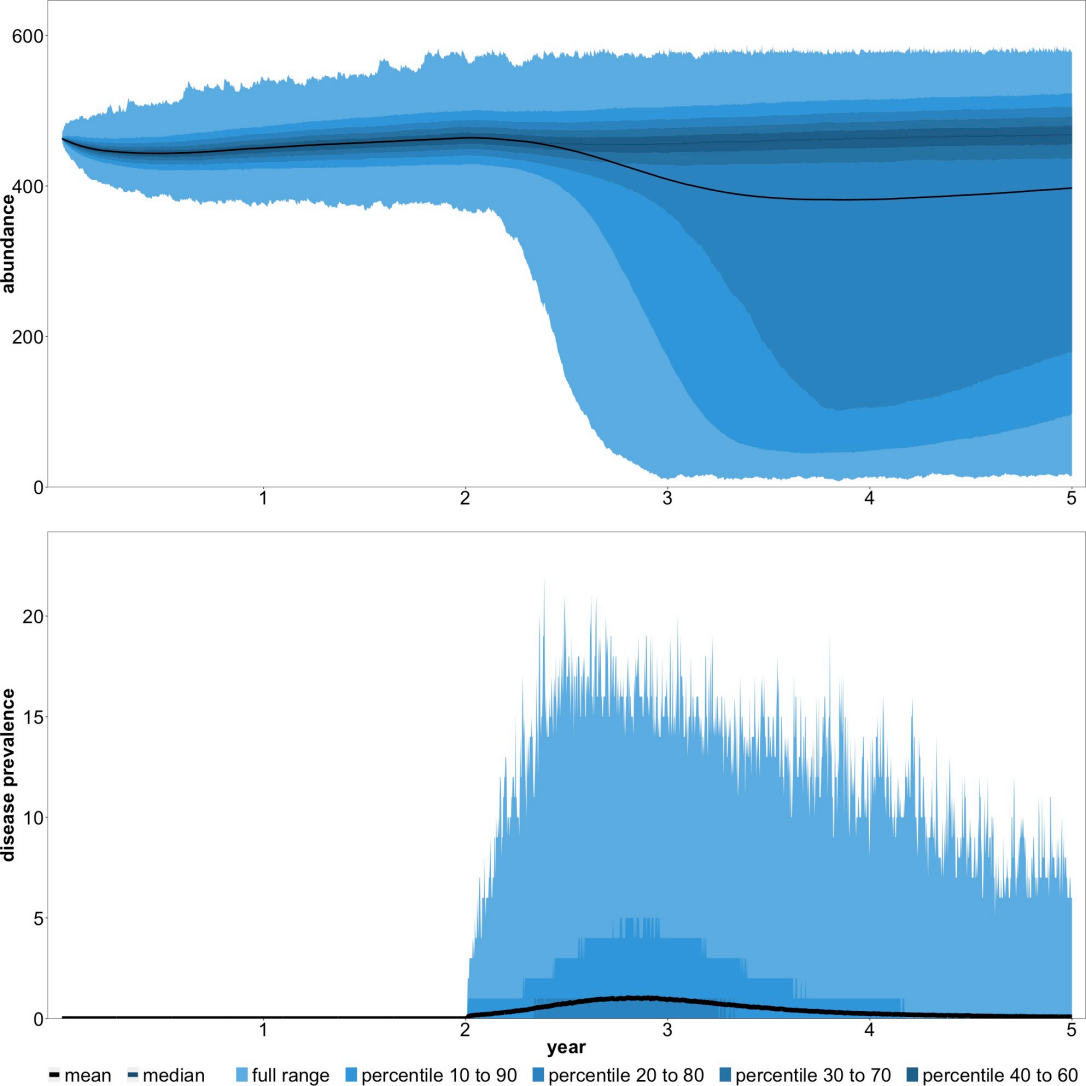
Abundance and rabies cases with no vaccination coverage and no management.

Before addressing the questions of puppy vaccination and female sterilization, we investigated a number of baseline scenarios. In these scenarios, and in all others that we present, we relied on the script-based version of the model. First, we examined population and disease dynamics in the absence of any management (Fig 4). Rabies was introduced by a single infectious dog at the beginning of year three. To gain a clear understanding of the benefits of vaccination in other scenarios, we assumed no existing vaccination coverage. Thus, disease incidence should be higher than is currently observed in many areas of South Africa. Furthermore, it should be noted that disease incidence is likely to be higher than indicated by Eq 1 with vaccination coverage set to zero. If Eq 1 is reasonable for all of South Africa, we would expect maximum disease incidence during an outbreak in a smaller area to be substantially higher.

The key result from the no management scenario was 497 average dog-days of infection. Note that we define one dog-day of infection as a single dog being infectious for one day. As an example, if an average of one dog is infectious on each day of a three-year period, the total dog-days of infection will be 1 x 3 x 365 = 1,095. Across 5,000 iterations of the simulation, maximum cases never exceeded a single dog in 62% of the iterations. In the iterations that we observed disease transmission to multiple dogs, the average maximum cases was 9. The total cost of rabies in this scenario was R43,126, which consisted entirely of PEP costs.

Next, we investigated a suite of baseline management scenarios in which we applied vaccination to juvenile and adult dogs (Table 3). Specifically, we defined three different total budgets for the five-year simulation period: R5,000, R20,000, and R40,000. We chose R40,000 as the largest budget because it resulted (approximately) in the typical recommendation of 70% vaccination coverage when puppies were included in the vaccination efforts. The other two budgets were included to explore the implications of varying degrees of budget limitation. At each total budget, we additionally examined annual, biennial, and reactive management. In the annual management scenarios, the budget was spread evenly over the entire five-year period; in the biennial scenarios, the budget was spread over years one, three, and five. The reactive scenarios assume that management only occurs once the disease is detected and continues to occur thereafter. Thus, in the reactive scenarios, the management budget was spread evenly over years three, four, and five.

**Table 3:** Baseline (only adult and juvenile vaccination) scenario results.

	dog-days of infection^1^	probability of outbreak^2^	max size of outbreak^3^	max vacc. coverage^4^	total cost^5^
**no management**	496.83	38%	9.07	0%	R43,126
**annual**					
**budget = R5,000**	78.65	26%	4	21%	R43,962
**budget = R20,000**	12.75	14%	2.58	45%	R58,264
**budget = R40,000**	5.16	7%	2.25	66%	R78,199
**biennial**					
**budget = R5,000**	63.35	28%	3.6	27%	R43,764
**budget = R20,000**	14.85	18%	2.66	58%	R58,261
**budget = R40,000**	8.28	12%	2.41	81%	R78,246
**reactive**					
**budget = R5,000**	101.12	36%	4.2	28%	R44,101
**budget = R20,000**	44.69	33%	3.61	60%	R58,519
**budget = R40,000**	37.52	34%	3.48	82%	R78,449

^1^ The average (across iterations) total (within iteration) number of dog-days of infection. One dog infective for one day equals one dog-day of infection.

^2^ The percent of iterations in which the maximum number of infective dogs on any day exceeds one.

^3^ The average (across iterations) maximum number of infective dogs on any day.

^4^ The average (across iterations) maximum percent of dogs on any day that have been vaccinated.

^5^ The average (across iterations) sum (within iteration) of all management cost and PEP costs (1 ZAR = 0.073 USD as of January 2019)

**Fig 5:**
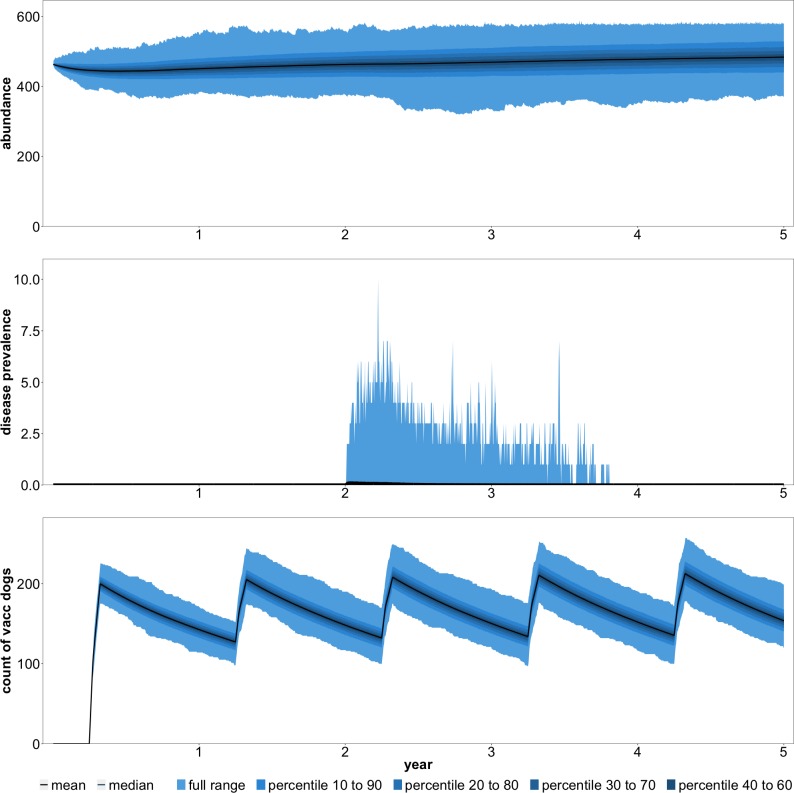
Abundance, disease cases, and vaccine coverage with annual vaccination (no puppies) and a total budget of R20,000.

The metric by which we judged the relative merits of the baseline strategies was dog-days of infection. This is equivalent to assuming the sole objective of management is to minimize the number of dog cases. We focused on dog-days of infection because it is the main driver of PEP costs and human mortality risk. Baseline results indicate that even very low levels of vaccination will substantially reduce rabies cases and burden (Fig 5). All three baseline scenarios with a budget of R5,000 resulted in an approximate 80% decrease in dog-days of infection. This alone was an important result. It suggested that low vaccination levels, insufficient for large scale elimination, still resulted in substantial reductions in human mortality risk.

Another key result of the baseline analyses was that annual vaccination campaigns were only preferred if the budget was sufficiently large. At the lowest budget (R5,000), the biennial strategy was superior to the annual and reactive strategies. There are several reasons for this. First, when resources were more concentrated in certain years (as in biennial), it pushed contact costs onto the steeper segments of the marginal cost curve. This means that it becomes costlier to find additional dogs to vaccinate. At low budget levels, this is less of a concern and it becomes more important to concentrate resources in years where rabies is actually present in the population. Second, annual management makes it more likely to re-contact dogs and apply a booster vaccine, which we assumed provides an additional three years of protection. But at low budgets, the chance of re-contacting dogs is likely to be low and this benefit would be mitigated. The preceding comparison notwithstanding, it was not our objective to provide guidance on management timing across years. These decisions often depend on factors other than cost-effectiveness. For example, annual vaccination in a local area may not be feasible given the resources available and the total size of the area under management. Additionally, reactive management may be required when a dog tests positive in an area that had been rabies-free for some time.

### Puppy vaccination

The observation that puppies respond well to rabies vaccination is not sufficient evidence to justify puppy vaccination during mass vaccination campaigns. There are two additional factors that should be considered. First, if the slope of the marginal contact cost curve is positive, it becomes costlier to contact additional dogs (on a per-dog basis) as a campaign continues. If the slope of the marginal cost curve is sufficiently steep, it suggests that puppies that happen to be contacted should be vaccinated because it will become increasingly costly to contact additional juveniles and adults. However, this must be weighed against the much higher mortality rate of puppies. In our target population, puppy mortality was nearly three times higher than adult mortality. Thus, even if a manager can forego a relatively small number of adult vaccinations and gain a relatively larger number of puppy vaccinations, mortality differences will cause the vaccinated puppies to exit the population earlier and potentially negate any advantage.

To investigate the optimality of puppy vaccination, we modified our baseline scenarios by vaccinating all puppies that are contacted. The results suggested that puppy vaccination reduced dog-days of infection and total disease cost under all management strategies (Table 4). This implied that the increasing cost of contacting dogs during campaigns outweighed the high puppy mortality in our study area. Although this might be an intuitive result in scenarios where vaccines are plentiful, we believe it is an important question when the management budget is insufficient to vaccinate a large percentage of the population. Finally, we once again found that, on a low budget, biennial campaigns were superior to annual and reactive campaigns.

**Table 4:** Puppy vaccination (also included adult and juvenile vaccination) results.

	dog-days of infection	probability of outbreak	max size of outbreak	max vacc. coverage	total cost^1^
**annual**					
**budget = R5,000**	69.75	25%	3.92	22%	R43,826
**budget = R20,000**	10.35	13%	2.49	49%	R58,247
**budget = R40,000**	4.67	6%	2.26	71%	R78,193
**biennial**					
**budget = R5,000**	61.27	27%	3.63	29%	R43,707
**budget = R20,000**	12.7	16%	2.57	63%	R58,276
**budget = R40,000**	7.28	10%	2.41	87%	R78,240
**reactive**					
**budget = R5,000**	99.51	37%	4.21	31%	R44,014
**budget = R20,000**	42.54	35%	3.42	65%	R58,504
**budget = R40,000**	36.86	35%	3.39	89%	R78,474

^1^1 ZAR = 0.073 USD as of January 2019

### Female sterilization

**Fig 6:**
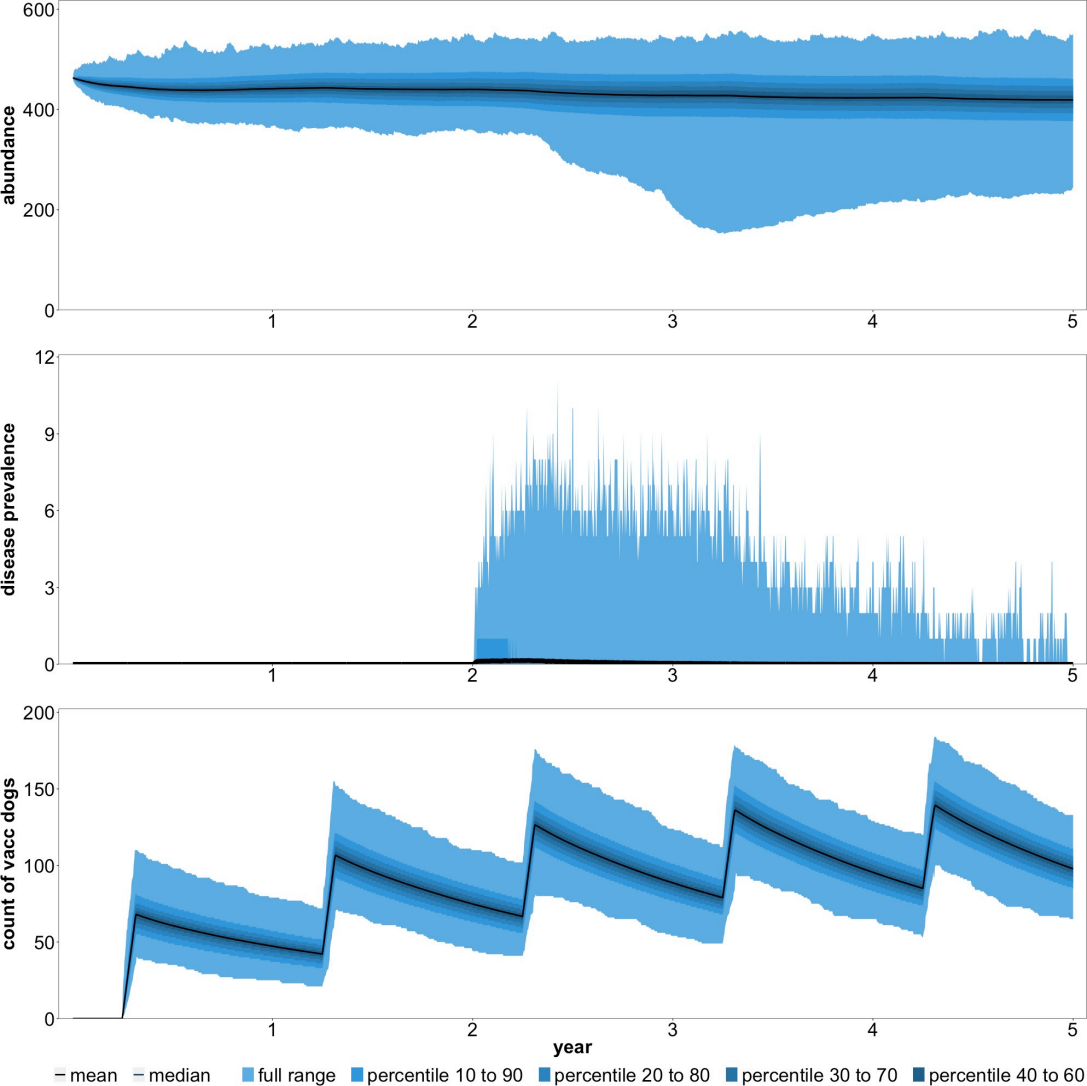
Abundance, disease cases, and vaccine coverage with annual vaccination (inc. puppies), female sterilization, and a total budget of R40,000.

In these scenarios, we assumed that any non-sterilized juvenile or adult female dog that was contacted during a vaccination campaign was surgically sterilized. Sterilization could be beneficial if reductions in population growth and turnover reduce future vaccination costs enough to justify not vaccinating some dogs in earlier periods. Based on the time period we examined, it was intuitive that sterilization would not effectively reduce the burden or total cost in the reactive scenario (Table 5). In the reactive scenario, management only occurs in response to an outbreak, before sterilization could slow growth and turnover. However, our findings suggested that sterilization under any strategy or budget was undesirable. In our study area, there was a constant influx of dogs from other areas, and sterilization had little effect on abundance even at high budget levels (Fig 6).

**Table 5:** Vaccination and female sterilization results.

	dog-days of infection	probability of outbreak	max prev. of outbreak	max vacc. coverage	total cost^1^
**annual**					
**budget = R5,000**	380.07	36%	7.84	6%	R46,613
**budget = R20,000**	149.46	30%	5.06	17%	R58,322
**budget = R40,000**	46.80	24%	3.53	31%	R76,151
**biennial**					
**budget = R5,000**	405.76	36%	8.03	7%	R46,905
**budget = R20,000**	183.02	32%	5.30	19%	R58,686
**budget = R40,000**	50.68	28%	3.45	34%	R76,145
**reactive**					
**budget = R5,000**	394.28	37%	8.01	9%	R46,875
**budget = R20,000**	218.66	36%	5.86	26%	R59,504
**budget = R40,000**	114.68	37%	4.38	45%	R77,861

^1^1 ZAR = 0.073 USD as of January 2019

To maximize the chances of female sterilization providing a net benefit, we examined a set of additional scenarios in which rabies was introduced at the beginning of the fifth year. We assumed a high budget in these scenarios so that marginal contact costs were relatively high. This increased the cost of vaccinating an additional dog relative to sterilizing females that were contacted earlier in vaccination campaigns. Furthermore, shifting the time of introduction back two years allowed more time for the benefits of sterilization to be realized. After four potential years of sterilization, abundance should be substantially lower. Despite these changes, vaccination-only was strongly preferred compared to campaigns that include sterilization.

### Sensitivity analysis

There were too many parameters to present a complete sensitivity analysis here, but an examination of results under alternative values of several important parameters was warranted. As discussed previously, the shape of the marginal contact cost curve has important implications. Higher and steeper marginal cost curves imply that capturing additional dogs during a campaign becomes increasing difficult and costly. This reduces the number of dogs that can be vaccinated, and makes it more likely that annual campaigns, puppy vaccination, and sterilization are part of an optimal strategy. To examine alternative contact costs, we specified two alternative cost curves that were 50% and 150% of the curve shown in Fig 2.

Results under the 50% and 150% alternative contact costs were qualitatively identical to the baseline contact costs with one exception (Table 6). Given a budget of R5,000 and annual campaigns, our results suggest that puppies should not be vaccinated when contact costs were 50% of the baseline. This is not a surprising result. Lower contact costs, annual campaigns, and a total budget of R5,000 ensure that contact costs remain very low even when only juveniles and adults are vaccinated. Thus, it is optimal to only vaccinate adults and juveniles because their mortality rate is lower.

**Table 6:** Sensitivity of results to alternative contact costs.

		contact costs relative to baseline^1^		
	budget	50%	100%	150%
puppy vaccination optimal? (with annual campaigns)	R5,000	No	Yes	Yes
	R20,000	Yes	Yes	Yes
	R40,000	Yes	Yes	Yes
annual campaigns optimal? (with puppy vaccination)	R5,000	No	No	No
	R20,000	Yes	Yes	Yes
	R40,000	Yes	Yes	Yes
female sterilization optimal? (with annual campaigns)	R5,000	No	No	No
	R20,000	No	No	No
	R40,000	No	No	No

^1^ Adult and juvenile vaccination only.

We additionally examined results under alternative values of the mean number of bites per rabid dog, which is the primary parameter that governs disease transmission (Table 7). Results were very similar, although a few important differences were noted. Unlike the baseline scenario, our results indicated that annual campaigns were optimal if the mean number of bites per rabid dog is set at either 50% or 150% of baseline. In the low-transmission (50%) and high-transmission (150%) scenarios, fadeout was more likely to occur before the fifth year. Thus, in these scenarios, any resources allocated to management in year five were likely to be wasted. Since annual management allocated less resources to year five, relative to biennial management, it was the optimal strategy at low budget when disease transmission was sufficiently low or high. The other difference relative to the baseline was the finding the biennial campaigns were preferred in the high-transmission scenario with a budget of R20,000. We were unable to precisely explain this result, although we suspected it was related to a complex interaction of marginal contact costs, vaccination rates, and disease transmission parameters. However, we cannot discount the possibility that it was an anomaly that resulted from an unlikely set of iterations.

**Table 7:** Sensitivity of results to alternative mean bites per rabid dog.

		mean bites relative to baseline^1^		
	budget	50%	100%	150%
puppy vaccination optimal? (with annual campaigns)	R5,000	Yes	Yes	Yes
	R20,000	Yes	Yes	Yes
	R40,000	Yes	Yes	Yes
annual campaigns optimal? (with puppy vaccination)	R5,000	Yes	No	Yes
	R20,000	Yes	Yes	No
	R40,000	Yes	Yes	Yes
female sterilization optimal? (with annual campaigns)	R5,000	No	No	No
	R20,000	No	No	No
	R40,000	No	No	No

^1^ Adult and juvenile vaccination only.

## Discussion

Our over-arching objective was to build a tool that can be used in applied settings to answer practical questions about how to best manage canine rabies in South Africa and elsewhere. The tool we provide is more accessible and more flexible than many of the other modeling tools for canine rabies management that we are aware of. Users can access the model easily and the interface allows many parameters to be tuned for the specific application. Furthermore, a wide variety of strategic options can be investigated. Any combination of vaccination, sterilization, contraception, and euthanasia can be specified, and these can be set specifically for each of six different demographic groups.

The results of our application suggested that, for dog populations with the ecological characteristics of our study population, puppies should be vaccinated during campaigns. Furthermore, our findings suggested that sterilization should not be included in vaccinations campaigns in these populations if the objective was to minimize the burden of rabies. However, this finding is likely dependent on the spatial scale of the vaccination efforts. In the relatively small population considered in our application, sterilization was not effective at limiting abundance because of the ongoing influx of dogs from other areas. If dogs in these other areas were also sterilized, the results could be different and would require a better understanding of factors driving in-migration of dogs. Finally, our findings suggested that, at relatively low budget levels, biennial vaccination campaigns were often preferable to annual campaigns. It should be noted that this interpretation assumed a fixed budget that could be shifted among years. In practice, this may not always be possible. In summary, our case study results suggested that puppies should be vaccinated, sterilization is not optimal if the spatial extent of management is not large (and perhaps not even then), and vaccination campaigns should be repeated annually in a scenario that assumed sufficient budget for such purpose.

There are several shortcomings of the model that users should be aware of. Although we have carefully parameterized the model based on published information and data we have collected, the suitability of these parameters for populations that differ substantially from the population we modeled in our application has not been tested. There are two specific concerns related to modeling large populations. First, the model we have built has no spatial detail other than a concept of dogs entering and exiting the population. In large populations with substantial spatial heterogeneity, the suitability of our model should be carefully considered. Second, because our model is a stochastic simulation model, it is slower than other types of models and computation time will increase approximately linearly with both the size of the population and the number of iterations specified. Finally, we acknowledge that we have not incorporated management actions related to awareness or education with the local population. We lacked data on these factors, but interested users could easily adjust model code to account for resulting differences (e.g. changes in contact costs and/or human bite rates).

Relative to other recently developed models of canine rabies management (e.g. [26]), our model has several key advantages. First, we have incorporated considerable sophistication and realism into economic and cost components of the model. Accounting for increasing marginal costs of contact or capture is of central importance when a manager is considering diverting resources to options such as fertility control, booster vaccination, or vaccination of puppies. Additionally, the ability to specify sex-specific fertility control costs and adjust these costs is important. Male and female sterilization are not equally costly, and chemo-sterilization is an evolving technology with sex-specific costs that are likely to change substantially as the technology is refined and becomes more widely available. Finally, our model is stochastic which is advantageous when judging the likelihood that a strategy will achieve a specific objective. Unlike a deterministic model, a stochastic model can be used to understand the tradeoffs between cost and the uncertainty of success.

Achieving the objective of “Zero by 30”–eliminating all human deaths from dog transmitted rabies–will require careful planning and efficient use of management resources. Efficient use of resources requires a broad range of strategic considerations, from the planning of multi-national efforts to the seasonal timing of campaigns at a specific location. Given that well-coordinated, large-scale management efforts are rare, there is immediate utility in a tool that helps managers maximize the benefits of resources allocated at the local level. The tool we developed can be applied to a targeted population to maximize the benefits of a specific budget allocation, or it can be used to estimate the minimum budget that would be needed to effectively manage rabies in a local dog population. In this way, available resources can be stretched to maximize the chance of achieving “Zero by 30”.
